# Colorectal Endoscopic Submucosal Dissection: Performance of a Novel Hybrid-Technology Knife in an Animal Trial

**DOI:** 10.3390/diagnostics13213347

**Published:** 2023-10-30

**Authors:** Jérémie Jacques, Horst Neuhaus, Markus D. Enderle, Ulrich Biber, Walter Linzenbold, Martin Schenk, Kareem Khalaf, Alessandro Repici

**Affiliations:** 1 Department of Hepato-Gastro-Enterology, University Hospital Center, 87042 Limoges, France; 2 Medical Clinic for Gastroenterology at the Hospital Duesseldorf, 40225 Duesseldorf, Germany; 3 Erbe Elektromedizin GmbH, 72072 Tuebingen, Germany; 4 Department of Experimental Medicine, University Hospital Tuebingen, 72076 Tuebingen, Germany; 5 Endoscopy Unit, Humanitas Clinical and Research Center IRCCS, 20089 Milan, Italy; 6 Department of Biomedical Science, Humanitas University, 20072 Milan, Italy

**Keywords:** endoscopy lower GI Tract, colorectal cancer, endoscopic resection, performance, complications

## Abstract

Endoscopic submucosal dissection (ESD) was developed for the removal of benign and early malignant lesions in the gastrointestinal tract. We aimed to evaluate the performance and safety of a novel high-pressure waterjet-assisted ESD knife in colorectal applications. Six female German Landrace pigs with an average weight of 62 kg (range 60–65 kg) were used in this prospective, randomized, and controlled study. Twenty-four ESDs were performed by three endoscopists: Twelve each with the new Erbe HYBRIDknife^®^ flex T-Type (HK-T) and the Olympus DualKnife^®^ J (DK-J), including six rectal and six colonic ESDs per instrument. The order of performance was randomized regarding anatomic position and instrument. As the primary endpoint, ESD knife performance characteristics were combined and rated on a 5-point Likert scale, with 5 Likert points (LP) representing the best response (5 = very good). The HK-T was rated significantly better than the DK-J (4.7 LP versus 4.4 LP, *p* = 0.0295), mainly because of HK-T injection ability (5 LP versus 3 LP, *p* < 0.0001) and hemostasis (5 LP versus 4 LP, *p* = 0.0452). There was no difference in procedure time (HK-T: 35 min versus DK-J: 34 min, *p* = 0.8005), resection diameter (3.1 cm versus 2.8 cm, *p* = 0.3492), injection volume (41 mL versus 46 mL, *p* = 0.5633), and complication rates. HK-T is as effective as DK-J in colorectal ESD in terms of dissection quality but has better injection and hemostatic properties. The impact of these technical advantages on the ESD treatment of patients with large superficial colorectal lesions remains to be clinically verified.

## 1. Introduction

ESD is a minimally invasive technique developed for endoscopic en-bloc removal of benign and early malignant gastrointestinal (GI) lesions [1,2,3,4]. It is the gold standard for the treatment of large (>2 cm in diameter) superficial/flat GI neoplasms involving approximately the first third of the submucosal layer [5,6,7,8,9,10,11]. Despite its advantageously low recurrence rates [6,10,12,13,14,15,16,17,18,19,20,21], colorectal ESD is difficult to establish in practice because it requires a high level of expertise associated with a long and shallow learning curve. In ESD, both the mucosa and submucosa are removed. To accomplish this, it may initially be helpful to mark the margins of the lesion to be removed with superficial cautery marks, except in the colon where margins can be easily identified. In addition, for en-bloc resections of lesions larger than 2 cm in diameter, a dedicated ESD knife can play a crucial role in procedural success. The HybridKnife^®^ is a unique technology combining an ultrafine high-pressure waterjet with an electrosurgical instrument designed for upper GI ESD procedures. It has been utilized in clinical trials [22,23,24,25] and evaluated in animal studies [26,27,28]. This flexible instrument allows a submucosal injection to create a safety cushion of saline in the submucosa at a preselected and high pressure through a standard working channel, reducing procedure time [29] and perforation rate [28,30]. The HybridKnife is also used for marking the targeted lesion, circumferential cutting, submucosal dissection, and coagulation, making it the only device available to perform the entire ESD procedure without exchanging multiple devices [25,31,32,33]. Colorectal lesions are comparatively difficult to treat due to the very thin intestinal wall and other anatomic difficulties [34,35,36,37,38,39]. Despite its theoretical advantages, the HybridKnife is not extensively used in colorectal ESD. The thickness and stiffness of the probe are not compensated by the high-pressure injection and are obstructive in the colorectum, where accuracy is critical due to the very narrow cutting plane.

**Figure 1:**
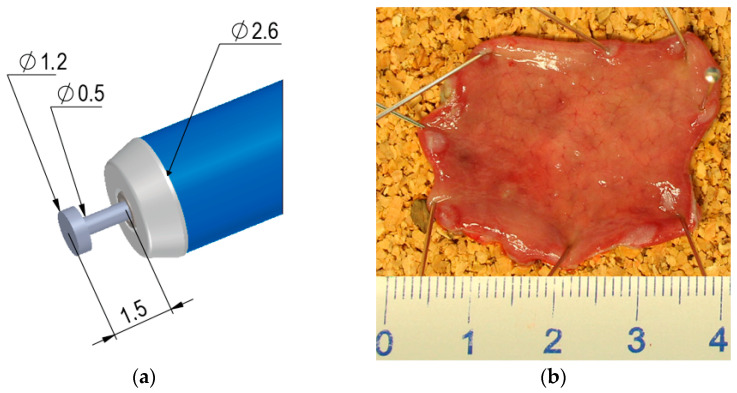
(**a**) Novel endoscopic submucosal dissection (ESD) instrument HYBRIDknife flex T-Type (Erbe Elektromedizin) with a 1.5-mm electrode length and an axial design (dimensions in mm, a second variant with 2.0-mm electrode length is available). A central capillary can deliver an ultrafine waterjet with a pressure of up to 80 bar; (**b**) an example of a rectal ESD resectate for ex vivo evaluation: the resection diameter was calculated from the area described by the circumference of the resectate (here: 8.22 cm² corresponding to a diameter of ~3.24 cm).

Against this background, a new hybrid knife for colorectal ESD was developed in close collaboration between clinical experts and engineers. This novel knife, the HYBRIDknife flex T-Type (HK-T, Figure 1a), combines the advantages of the current HybridKnife with a thinner electrode that maintains a stable position and is therefore particularly suitable for colorectal lesions. The HK-T is also more flexible, which could be especially beneficial for procedures performed in a retroflex position. The HK-T has an axial design and an electrode length of 1.5 mm when projected. An ultrafine waterjet can be delivered through a central capillary at the tip with a pressure of up to 80 bar. The “T” in T-Type describes the shape of the electrode, which has a disc-shaped formation at its tip. There are also variants with 2.0 mm electrode length and a needle-shaped electrode, the I-Type. The aim of this in vivo animal study was to evaluate the performance and safety of the HK-T in colorectal applications.

## 2. Materials and Methods

In accordance with the “Guide for the Care and Use of Laboratory animals” published by the National Academy of Sciences, this study was reviewed and approved by the institutional review board for animal experiments, Regional Council Tübingen, Germany (approval no. C01/17). All investigations were in accordance with the EU directive 2007/526/EG and the EU guideline 2010/63/EU. Six female German Landrace pigs (Sus scrofa domesticus) aged approximately 12 weeks with a mean body weight of 62 ± 2.3 kg (range 60–65 kg) were used. The animals were obtained from a commercial pig breeder in Baden-Wuerttemberg, Germany, and were identified with ear tags. They were kept in cages at a temperature of approximately 20 °C and a relative humidity of approximately 60% with natural day–night cycles. They received a standard diet, with tap water as needed. Four days prior to the intervention, the animals were restricted to a liquid diet. The pigs were deprived of food, but water consumption was allowed for 24 h before the procedure. To ease bowel preparation, a laxative was administered on the morning of the intervention day. The animals were medicated and anesthetized according to the following protocol: Premedication was administered by intramuscular injection of atropine (0.05 mg/kg), azaperone (4.0 mg/kg; Stresnil^®^, Elanco GmbH, Cuxhaven, Germany), ketamine (14.0 mg/kg, Ketaminol^®^, Intervet Deutschland GmbH, Unterschleißheim, Germany), and midazolam (1.0 mg/kg; Midazolam-hameln, Hameln pharma GmbH, Hameln, Germany). Before tracheal intubation, a bolus of 2.0–5.0 mg/kg propofol (Propofol MCT Fresenius, Fresenius Kabi Deutschland GmbH, Bad Homburg, Germany) was given intravenously. During the intervention, the animals were ventilated and kept under deep anesthesia by isoflurane (0.8–1.6 vol%, Isofluran CP^®^, CP-Pharma Handelsgesellschaft mbH, Burgdorf, Germany) and for analgesic coverage by fentanyl (Fentanyl-ratiopharm^®^, ratiopharm GmbH, Ulm, Germany) at an intravenous dose of 30–100 µg/kg/h. In addition, butylscopolamine (0.5 mg/kg, Buscopan^®^, Boehringer Ingelheim Pharma GmbH, Ingelheim am Rhein, Germany) was administered immediately prior to the intervention to inhibit peristaltic movements of the colorectum. Colonic irrigation was performed with tap water before the intervention. The intraoperative monitoring of the animal included ECG, pulse oximetry, and capnometry. At the end of the intervention, the animals were painlessly euthanized with a lethal dose of the T61 solution (Intervet Deutschland GmbH, Unterschleißheim, Germany).

This was a randomized, controlled, and prospective in vivo animal study. Twenty-four colorectal ESD procedures were performed by three endoscopists (the authors J.J., A.R., and H.N.). All participants had performed ESD procedures for more than 10 years. To identify possible improvements of the HK-T ESD system, this system was considered as the test group (Video S1) and the DK-J ESD system as the control group, each of which was assigned twelve ESD procedures, including six rectal and six colonic ESD procedures. The procedures were performed in six pigs, i.e., two ESD procedures with HK-T and DK-J in each pig in direct comparison, and each endoscopist performed the interventions in two animals. Consequently, each endoscopist performed two ESDs per anatomic position and instrument, i.e., a total of eight ESDs. The order of performance was randomized with respect to the anatomic position (rectum and colon) and instrument (HK-T and DK-J). The HK-T (Figure 1a) and DK-J ESD knives were part of ESD systems consisting of an ESD knife, an electrosurgical/hydrosurgical supply unit, and corresponding generator algorithms and settings (Table 1).

Endoscopists were instructed to perform colorectal en-bloc ESDs with a minimum diameter of 2 cm. Rectal ESDs were performed up to a distance of approximately 20 cm from the anal verge. Colonic ESDs were performed at a distance greater than 20 cm and up to 40 cm from the anal verge. First, the endoscope was placed in the desired position, and then the instrument was inserted. Instrument insertion, marking of artificial lesion margins with coagulation points, elevation of the mucosa by waterjet/needle injection, circular incision and dissection of the submucosa, coagulation of blood vessels during/after resection if necessary, and finally retrieval of the resectate comprised a single colorectal ESD procedure. A short transparent cap was attached to the tip of the endoscope to allow continuous endoscopic vision and to apply tension to the connective tissue for submucosal dissection. The DK-J system requires the use of an endoscopic injection needle for initial mucosal puncture before submucosal cushioning, whereas the HK-T system uses a high-pressure waterjet. In this regard, the instruments were changed only at the request of the endoscopist.

After each ESD procedure, the performance of the ESD systems was evaluated by the endoscopist using a 5-point Likert scale. The meanings of the Likert points (LP) were assigned as follows: 1 = very poor, 2 = poor, 3 = neutral, 4 = good, and 5 = very good. ESD system performance was characterized by ease of instrument insertion, coagulation mark visibility, injection ability, mucosal cutting performance, submucosal dissection performance, hemostatic capability, instrument flexibility, electrode length stability, suction performance with instrument inserted, electrode/operation field visibility, and working speed. The primary outcome, ESD knife performance, was calculated per instrument per endoscopist as the average of the corresponding 17 Likert items (Table 2) obtained per ESD. Before the start of the procedures, each endoscopist was informed of the upcoming interview so that the relevant performance characteristics and the type of interview were known in advance. Procedure time was defined as the time in minutes between the first marking around the artificial lesion and the end of dissection. Complications, i.e., minor bleeding and major bleeding requiring the use of hemostatic forceps, and perforations were counted during the procedures. Similarly, the total injection volume in milliliters via the waterjet system and, if used, the syringe used in combination with the endoscopic injection needle was measured. After the procedure, the en-bloc resection was confirmed or not, and the resectate was pinned on a cork and photographed with a millimeter gauge (Figure 1b). Later, the resection diameter was calculated from the dissection area in mm^2^, which was described by the circumference of the resectate. A laboratory microscope software was used for this purpose. The dissection speed was defined as the dissection area divided by the procedure time.

GraphPad Prism version 7.05 (GraphPad Software, San Diego, CA 92108, USA) was used for the statistical analyses. Descriptive statistics (mean, standard deviation, median, and interquartile range) were performed to describe the basic characteristics of the collected data. The results of combined/averaged Likert items (measure of ESD knife performance) were continuous and normally distributed according to a KS-Test. Therefore, differences between the two groups were determined using an unpaired one-tailed Student’s *t*-test to identify possible improvements in the HK-T ESD system. Scores on individual Likert items (a measure of individual ESD performance aspects) are categorical and were likewise compared with a one-tailed Mann-Whitney rank test. The normal distribution of other continuous outcomes was also evaluated with a KS Test. Differences were determined with an unpaired two-tailed Student’s *t*-test for normally distributed data or with the Mann–Whitney-U-Test for non-normally distributed data. *p*-values < 0.05 were considered statistically significant. No explicit sample size calculation was performed. Instead, a sample size of 12 per group was chosen as a rule of thumb for a pilot study [40].

## 3. Results

### 3.1. Baseline Data: En-Bloc Resection Rate, ESD Size and Injection Volume

All 24 ESD procedures were completed in one piece, i.e., the *en-bloc* resection rate was 100%. We found no significant differences in ESD size between the two ESD systems (Table 3, HK-T: 3.1 ± 1.0 cm versus DK-J: 2.8 ± 0.5 cm, *p* = 0.3492) and no difference between rectal and colonic ESD size (Table 3, rectum: 3.1 ± 0.7 cm versus colon: 2.8 ± 0.9 cm, *p* = 0.2892). The ESD diameter ranged from 1.5 cm to 4.7 cm, and only one ESD in the colon with HK-T was smaller than the minimum 2 cm diameter specified. The injection volume was not significantly different between the two ESD methods (HK-T: 41 ± 13 mL versus DK-J: 46 ± 27 mL, *p* = 0.5633). However, colonic ESDs received, on average, 20 mL more saline than rectal ESDs (Table 3, rectum: 33 ± 13 mL versus colon: 53 ± 23 mL, *p* = 0.0147). In addition, no significant differences were observed in ESD size and injection volume between the two ESD systems within the rectal and colonic ESD procedures (Table 3).

### 3.2. Primary Endpoint: ESD Knife Performance

**Figure 2:**
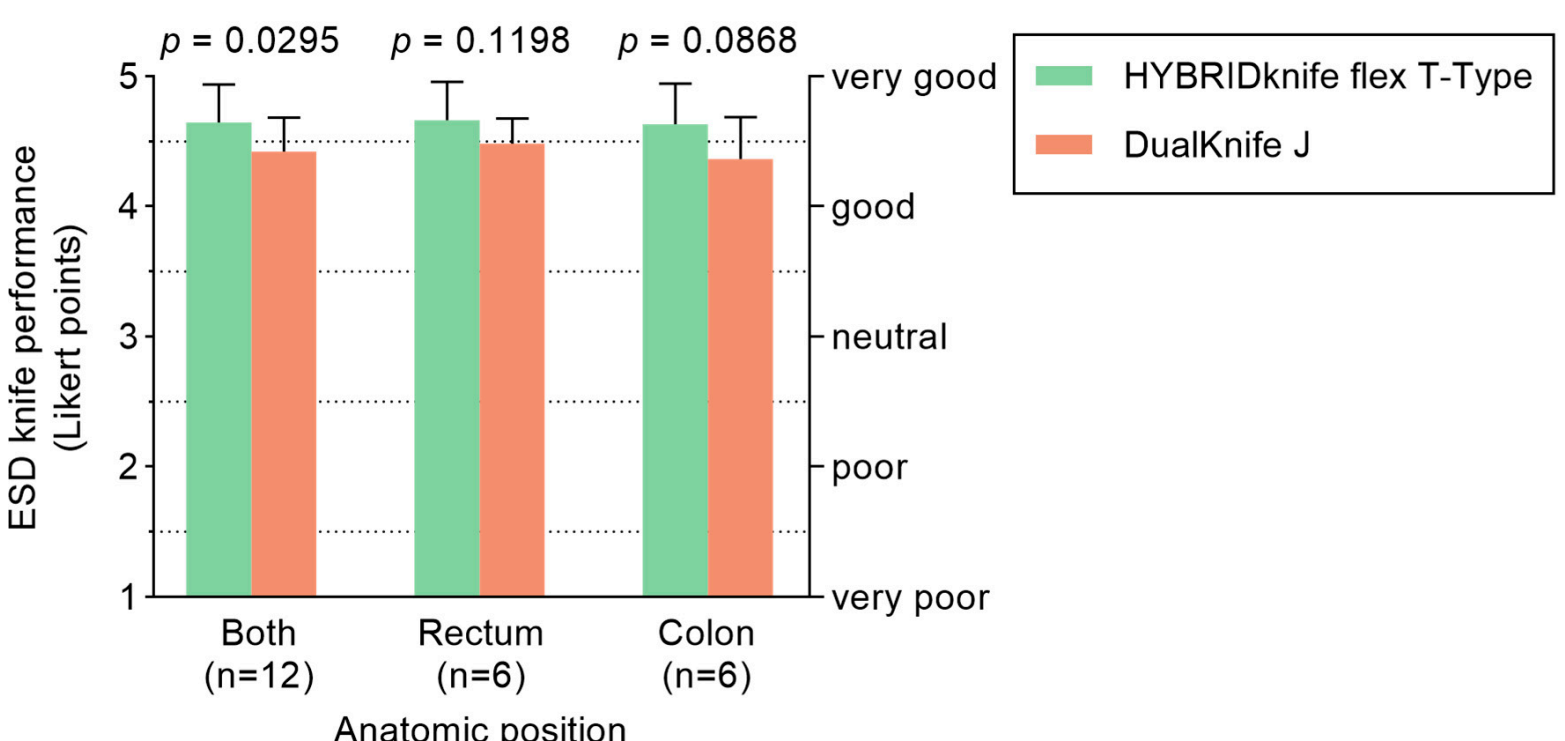
Grand average of instrument performance (error bars show SD) from 17 individual performance characteristics of HYBRIDknife flex T Type (green) and DualKnife J (orange). Three experienced endoscopists for colorectal endoscopic submucosal dissection were included in the evaluation.

**Figure 3:**
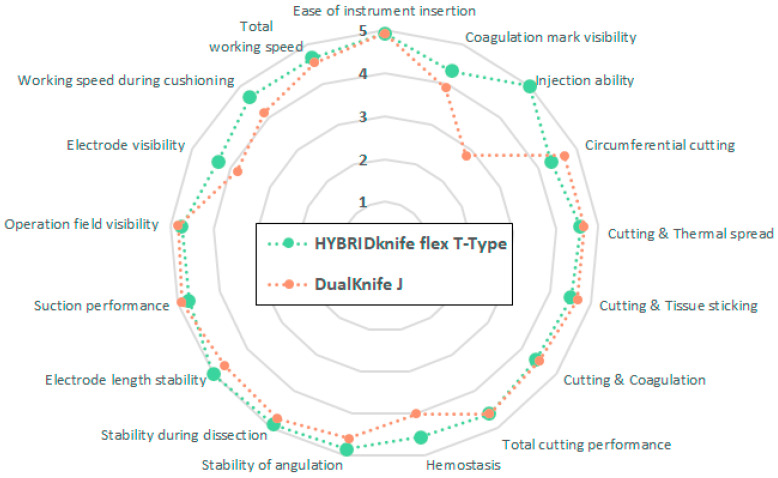
Individual mean performance scores of HYBRIDknife flex T-Type (green) and DualKnife J (orange) evaluated by three experienced endoscopists for colorectal endoscopic submucosal dissection. 5 = very good; 4 = good; 3 = neutral; 2 = poor; 1 = very poor.

The mean ESD performance score (Figure 2) of the HK-T (4.7 ± 0.29 LP) was significantly better (*p* = 0.0295) than that of DK-J (4.4 ± 0.26 LP). This difference was not significant in subgroup analyses of rectal (4.7 ± 0.30 LP versus 4.5 ± 0.19 LP; *p* = 0.1198) and colonic ESDs (4.6 ± 0.31 LP versus 4.4 ± 0.32 LP; *p* = 0.0868). However, colonic ESDs showed a statistical tendency in favor of HK-T (*p* = 0.0868). Evaluation of the individual performance aspects of both ESD knives (Figure 3) revealed significant advantages of HK-T in injection ability (medians HK-T: 5 LP versus DK-J: 3 LP, *p* < 0.0001) and hemostatic property (medians HK-T: 5 LP versus DK-J: 4 LP, *p* = 0.0452). In addition, a non-statistically significant difference was seen for HK-T in terms of coagulation mark visibility (medians HK-T: 5 LP versus DK-J: 4 LP, *p* = 0.0758), working speed during submucosal cushioning (medians HK-T: 5 LP versus DK-J: 4 LP, *p* = 0.0800), and electrode visibility (medians HK-T: 5 LP versus DK-J: 4 LP, *p* = 0.0949).

### 3.3. Secondary Endpoints

Procedure time was not significantly different between HK-T (35 ± 11 min) and DK-J (34 ± 8.7 min, *p* = 0.8005), as was dissection speed (HK-T: 25 ± 15 mm²/min versus DK-J: 20 ± 9.3 mm²/min, *p* = 0.3751). There was also no significant difference between rectal and colonic ESDs in terms of procedure time (rectum: 35 ± 12 min versus colon: 34 ± 7.1 min, *p* = 0.8925) and dissection speed (rectum: 24 ± 9.9 mm²/min versus colon: 20 ± 15 mm²/min, *p* = 0.4496). Dissection speed correlated positively with ESD size (r² = 0.67, *p* < 0.0001), and consequently, procedure time was not a function of ESD size (r² = 0.06, *p* = 0.2572) in the given diameter range. Perforation rate was not significantly different between instruments (Table 4, HK-T: 2/12 versus DK-J: 3/12, *p* > 0.99), but perforations occurred exclusively in the colon (rectum: 0/12 versus colon: 5/12, *p* = 0.0373). The rate of minor bleeding was not significantly different between the knives (HK-T: 4/12 versus DK-J: 6/12 *p* = 0.6802), as was the rate of major bleeding requiring the use of hemostatic forceps (HK-T: 0/12 versus DK-J: 1/12, *p* > 0.99). The rate of minor (rectum: 6/12 versus colon: 4/12, *p* = 0.6802) and major bleeding (rectum: 1/12 versus colon: 0/12, *p* > 0.99) did not differ significantly between anatomic positions. Furthermore, no significant differences were observed for these complications between the two ESD systems within the rectal and colonic ESD procedures (Table 4).

## 4. Discussion

The hybrid knife product family is designed for elevation, irrigation, dissection, and preparation of tissue layers by high-pressure waterjet application and monopolar electrosurgical cutting and coagulation. Like the new HK-T system, the original HybridKnife system allows simultaneous elevation and dissection, which shortens the operation time [29]. The pressure of the waterjet can be regulated according to the lesion, tissue layer, and application site. This allows a clear view of the operation site [34]. This randomized, controlled, and prospective animal study demonstrated that ESD performance scores were significantly better with the new HK-T system than with the DK-J system. This improvement was mainly because the HK-T system showed superior injection ability and hemostatic properties. A non-statistically significant difference was seen for the HK-T in terms of coagulation mark visibility, working speed during submucosal cushioning, and electrode visibility. Technically, the high-pressure injection of the HK-T is an advantage over the DK-J, which is particularly helpful in reinjecting the cushion during the course of the ESD procedure without changing instruments and explains the perceived higher working speed during submucosal cushioning. The improved hemostasis is most likely due to the T-Type disc-shaped electrode at the probe tip, which provides a larger contact area (diameters of 1.2 mm versus 0.65 mm) and thus greater hemostatic capacity, also resulting in better electrode and coagulation mark visibility.

Resection size, injection volume, and complication rates did not differ significantly between the groups. In addition, there was no significant difference in procedure time between the two ESD knives. Despite the fact that the HK-T was perceived slightly faster in terms of submucosal cushioning, the procedure time per ESD was consistent at approximately 35 min. In contrast, the procedure time per ESD for early gastric cancer is significantly shorter with the waterjet-assisted technique [29]. This advantage does not appear to translate directly to colorectal ESD. The average procedure time was similar to that reported for a conventional group in early gastric cancer [29]. However, we found that the raw dissection speed values were better with HK-T by an average of 5 mm²/min. The test statistic was not significant (two-tailed *p* = 0.3751; one-tailed *p* = 0.1876), but the post-hoc-calculated statistical power was just 14%. Statistically demonstrating a 5-mm²/min (20%) increase in speed, given the unexpectedly high observed pooled standard deviation of 12.4 mm²/min, requires a sample size of 77 ESDs per group, assuming the superiority of one instrument (one-tailed *t*-test), an alpha of 5%, and statistical power of 80%.

The new ESD technique with HK-T, as demonstrated here in the porcine model, could be equally effective in the human clinical setting, although the thinner colon wall of the pig [41] and more fatty tissue in the submucosa with many blood vessels [28] must be considered. Consequently, the perforation rate in the colon was high (42%), which would not be acceptable in the clinical setting. However, it is known that ESD in the colon of pigs is much more difficult than in humans due to the thinner submucosal and muscular layers (muscularis propria). In a previous animal study, a colonic perforation rate of more than 20% was found, even in the hands of a Japanese expert [28]. Regarding the risk ratio of complications, we observed equal or lower risks of perforation and bleeding with HK-T compared with DK-J, suggesting improved safety with the use of the HK-T ESD system. However, because of the small sample size, these differences need to be further verified with a larger sample size in the clinical setting.

The strengths of this study are the prospective randomized controlled study design, the deep expertise of the endoscopists in the field of colorectal ESD, and the comparison with the DK-J, the most commonly used knife for colorectal ESD. The main limitations are the small sample size (n = 12 per group) and the restriction to animals. However, this is a pilot technical study to confirm the potential of a new ESD knife and to analyze outcome measures, as well as to better plan comparative human clinical studies with a more adjusted sample size. Because high-pressure waterjet injection compensates for fluid leakage during ESD and fluid leakage is directly related to the duration of the procedure and the size of the resectate, the fact that the experts resected predominantly smaller lesions of approximately 3 cm in diameter could be considered a limitation of the study and a disadvantage for the HK-T system. It could be suggested that high-pressure waterjet injection is preferable for larger lesions, where leakage of the injection fluid would hinder the completion of the procedure. In addition, experts work faster than trainees and nonexperts. The possibility of submucosal elevation by high-pressure waterjet injection would certainly help the latter. However, whether trainees benefit more from the use of the HK-T during the learning curve of ESD or other advanced endoscopic procedures would need to be demonstrated in more large-scale and clinical studies. The apparent small gain in overall performance with the HK-T (4.7 LP versus 4.4 LP) is also due to the fact that the technological maturity of ESD knives is already extremely high. In addition, because it was not practically possible to blind the endoscopists to the use of the two ESD systems, the performance evaluations could be biased given the small number of three endoscopists and their personal preferences. This may have been a disadvantage for the new HK-T system, as preferences evolve with time and the use of existing systems. Especially for the aspects of performance (Figure 3), for which there were no more than 12 evaluations per group by three endoscopists, the sample size was a significant limitation, and the 5-point Likert scale provided did not allow for very fine-grained individual responses. Nonetheless, improvements in individual aspects of performance can have significant clinical benefits, leading to improved patient care.

## Figures and Tables

**Table 1:** ESD systems and settings.

ESD Knife	Electrosurgical Unit	Modes/Settings
HYBRIDknife flex T-Type (HK-T) cutting knife length: 1.5 mm when projected; working length: 2300 mm	Erbe VIO^®^ 3 (REF 10160-000, software version: 1.1.6) + ERBEJET^®^ 2 (REF 10150-000, software version: 1.1.5)	Marking of artificial lesion: forcedCOAG Effect 1.0–3.0
		Incision: endoCUT^®^ I Effect 2, cutting duration 3 (100 ms), cutting interval 3 (720 ms)
		Dissection: swiftCOAG Effect 4.0–6.0 (120 W–153 W)
		Injection: ERBEJET^®^ 2: Effect 15–30 + endoscopic injection needle (REF NM-412L-0423) if indicated
		Coagulation: swiftCOAG Effect 4.0–6.0 (120 W–153 W) or softCOAG^®^ Effect 5.0 (90 W)
Olympus DualKnife J (DK-J) KD-655U; cutting knife length: 1.5 mm when projected; working length: 2300 mm	Erbe VIO^®^ 3 (REF 0160-000, software version: 1.1.6) + Erbe EIP 2 (REF 10325-000)	Same as for HK-T but with endoscopic injection needle using EIP 2 for injection: 30–80%

ESD = Endoscopic submucosal dissection

**Table 2:** Likert items for evaluation of ESD knife performance.

Performance Characteristic	Statement
Ease of instrument insertion	The insertion of the instrument is completely smooth and without resistance.
Coagulation mark visibility	The coagulation marks are effectively visible.
Injection ability	The injection ability of the instrument is optimal.
Circumferential cutting	The instrument is ideal for circumferential cutting.
Cutting & Thermal spread	The cutting in terms of thermal spread is optimal.
Cutting & Tissue sticking	The cutting in terms tissue sticking is optimal.
Cutting & Coagulation	The cutting in terms of coagulation is optimal.
Total cutting performance	The overall cutting performance is optimal.
Hemostasis	The hemostasis is optimal and needs no additional intervention.
Stability of angulation	The endoscope maintains the chosen deflection/angulation with inserted instrument.
Stability during dissection	The ability to smoothly move the instrument through the target tissue without mechanical tension while cutting is optimal.
Electrode length stability	The pre-set electrode length was stable throughout the procedure.
Suction performance	The suction works sufficiently with the inserted instrument.
Operation field visibility	The visibility of the operation field was not impaired by the instrument.
Electrode visibility	The electrode was consistently visible during the procedure.
Working speed during cushioning	The working speed during submucosal cushioning was optimal.
Total working speed	The overall working speed for the entire ESD procedure was optimal.

**Table 3:** Baseline data of colorectal ESD procedures with HYBRIDknife flex T-Type (HK-T) and DualKnife J (DK-J).

	ESD Knife		*p* Value
	HK-T (*n* = 12)	DK-J (*n* = 12)	(*t*-test)
ESD diameter, mean ± SD (range), cm	3.1 ± 1.0 (1.5–4.7)	2.8 ± 0.5 (2.1–3.6)	0.35
Rectum, *n* (%)	6 (50)	6 (50)	
Colon, *n* (%)	6 (50)	6 (50)	
Injection volume, mean ± SD (range), mL	41 ± 13 (22–64)	46 ± 27 (18–105)	0.56
	**Anatomic position**		
	**Rectum (*n* = 12)**	**Colon (*n* = 12)**	
ESD diameter, mean ± SD (range), cm	3.1 ± 0.7 (2.3–4.4)	2.8 ± 0.9 (1.5–4.7)	0.29
HK-T, *n* (%)	6 (50)	6 (50)	
DK-J, *n* (%)	6 (50)	6 (50)	
Injection volume, mean ± SD (range), mL	33 ± 13 (18–64)	53 ± 23 (32–105)	0.0147
	**ESD knives in rectum**		
	**HK-T (*n* = 6)**	**DK-J (*n* = 6)**	
ESD diameter, mean ± SD (range), cm	3.5 ± 0.8 (2.3–4.4)	2.8 ± 0.4 (2.3–3.4)	0.11
Injection volume, mean ± SD (range), mL	39 ± 15 (22–64)	27 ± 8.1 (18–36)	0.14
	**ESD knives in colon**		
	**HK-T (*n* = 6)**	**DK-J (*n* = 6)**	
ESD diameter, mean ± SD (range), cm	2.8 ± 1.1 (1.5–4.7)	2.8 ± 0.7 (2.1–3.6)	0.96
Injection volume, mean ± SD (range), mL	42 ± 11 (32–58)	64 ± 28 (33–105)	0.10

ESD = Endoscopic submucosal dissection; SD = Standard deviation of the mean.

**Table 4:** Intraoperative complications.

Complication, Ratio (Percentage)	ESD Knife		*p* Value	Risk Ratio
	HK-T (*n* = 12)	DK-J (*n* = 12)	(Fisher’s Test)	(HK-T/DK-J)
Perforation	2/12 (17)	3/12 (25)	>0.99	0.67
Minor bleeding	4/12 (33)	6/12 (50)	0.6802	0.67
Major bleeding	0/12 (0)	1/12 (8)	>0.99	0.00
	**Anatomic position**			
	**Rectum (*n* = 12)**	**Colon (*n* = 12)**		
Perforation	0/12 (0)	5/12 (42)	0.0373	n/a
Minor bleeding	6/12 (50)	4/12 (33)	0.6802	n/a
Major bleeding	1/12 (8)	0/12 (0)	>0.99	n/a
	**ESD knives in rectum**			
	**HK-T (*n* = 6)**	**DK-J (*n* = 6)**		
Perforation	0/6 (0)	0/6 (0)	>0.99	-
Minor bleeding	2/6 (33)	4/6 (67)	0.5671	0.50
Major bleeding	0/6 (0)	1/6 (17)	>0.99	0.00
	**ESD knives in colon**			
	**HK-T (*n* = 6)**	**DK-J (*n* = 6)**		
Perforation	2/6 (33)	3/6 (50)	>0.99	0.67
Minor bleeding	2/6 (33)	2/6 (33)	>0.99	1.00
Major bleeding	0/6 (0)	0/6 (0)	>0.99	-

ESD = Endoscopic submucosal dissection; HK-T = HYBRIDknife flex T-Type (Erbe); DK-J = DualKnife J (Olympus).

## Data Availability

The data presented in this study are available upon reasonable request from the corresponding author.

## Supplementary Materials

The following supporting information can be downloaded at: https://www.mdpi.com/article/10.3390/diagnostics13213347/s1, Video S1: Exemplary video of the performance of a rectal ESD in the pig.
